# Anesthesia for mandibular yolk sac tumor in children after radiotherapy and chemotherapy: A case report

**DOI:** 10.1002/ccr3.6481

**Published:** 2022-11-15

**Authors:** Shuiting Zhang, Liang Wen, Hui Zou, Shaosan Liu, Ruping Dai

**Affiliations:** ^1^ Department of Anesthesiology, The Second Xiangya Hospital Central South University Changsha China; ^2^ Anesthesia Medical Research Center Central South University Changsha China; ^3^ Clinical Nursing Teaching and Research Section, The Second XiangYa Hospital Central South University Changsha China

**Keywords:** bronchoscope intubation, children, spontaneous ventilation, yolk sac tumor

## Abstract

Yolk sac tumor (YST), also known as endodermal sinus tumor, is a malignant germ cell tumor that usually affects children aged >3 years. It is commonly observed in the gonadal sites (testis or ovary) but is extremely rare in the mandibular regions. This study describes the anesthesia process for spontaneous ventilation bronchoscope intubation in the rare case of a 7‐year‐old child with extragonadal primary YST of the face, refractory to radiotherapy, and chemotherapy.

## 1 INTRODUCTION

Yolk sac tumor (YST) is a germ cell tumor subtype that occurs in newborns and infants, as well as young adults aged 14–44 years. It is rarely found primarily in the extragonadal sites with no involvement of the gonads.^1^ We describe a case of primary maxillofacial YST refractory to radiotherapy of the head and neck and various cycles of systemic chemotherapy in a patient with restricted mouth opening. Currently, no cases of induction of anesthesia in children with YST have been reported. This report could help bolster the knowledge of anesthesiologists dealing with these uncommon tumors.

## 2 CASE REPORT

### 2.1 Basic information

**FIGURE 1:**
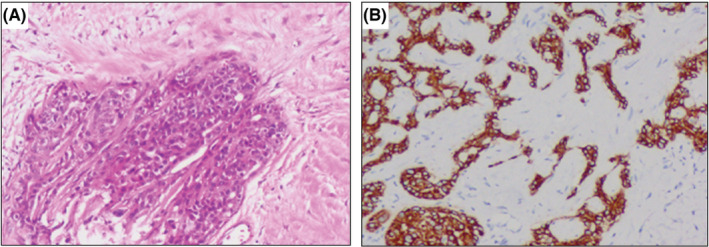
Histopathology of the endometrial yolk sac tumor. (A, B) H&E staining analysis (A) and immunohistochemical staining of SALL4 (B, positive) in tumor tissue.

**FIGURE 2:**
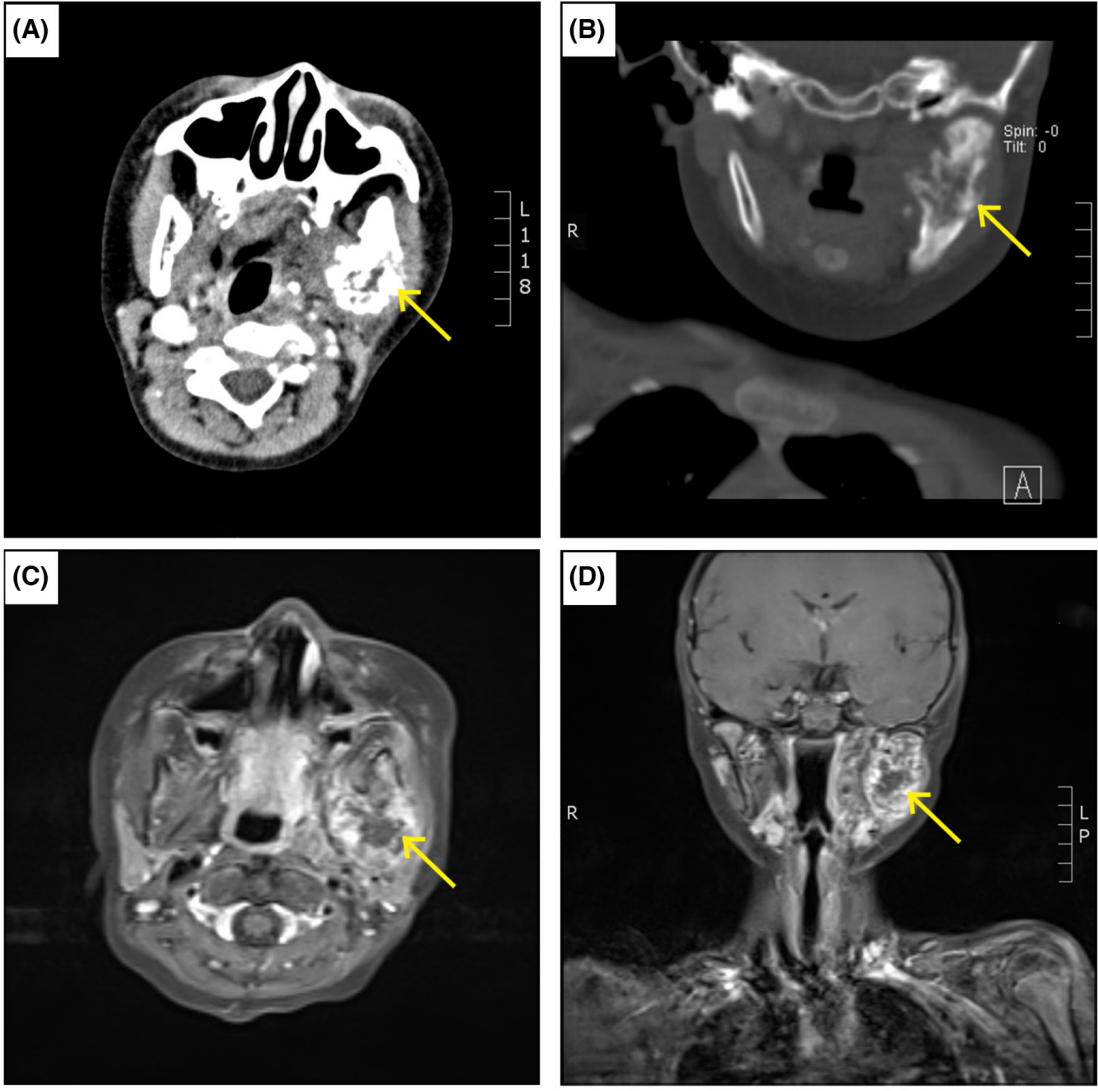
Radiographic images of the yolk sac tumor patient. (A, B) Computed tomography (CT) showed a left facial mass (Yellow arrow) with transverse (A) and coronal section (B). (C, D) Magnetic resonance imaging (MRI) displaced an abnormal signal mass in the left facial mass (arrow) with transverse (C) and coronal section (D).

A 7‐year‐old male child, weighing 24 kg, presented with a painful swelling on the left side of the face that increased rapidly in size 5 years ago. Results of the previously performed biopsy at Xiangya Hospital revealed a confirmed YST diagnosis. The patient then underwent six cycles of cisplatin, etoposide, and bleomycin (PEB) chemotherapy. However, the facial swelling recurred after 3 years. The pathological analysis was repeated (Figure 1), which revealed the same results as before. Subsequently, four cycles of PEB/etoposide, ifosfamide, and cisplatin chemotherapy were administered. Unfortunately, the facial swelling reappeared after 4 months, and PEB chemotherapy and radiotherapy were started. However, the size of the lesion did not decrease (Figure 2). Hence, extended resection of the facial mass under general anesthesia was planned.

Preoperative biochemical examination results, chest radiograph, and electrocardiogram of the patient showed no apparent abnormalities. Abdominal ultrasound examination for any abdominal mass or lymph nodes showed normal findings. Preoperative evaluation of the patient revealed restricted mouth opening (Mallampati classification IV), limited receding of the head, and a short neck.

### 2.2 Induction of anesthesia

**FIGURE 3:**
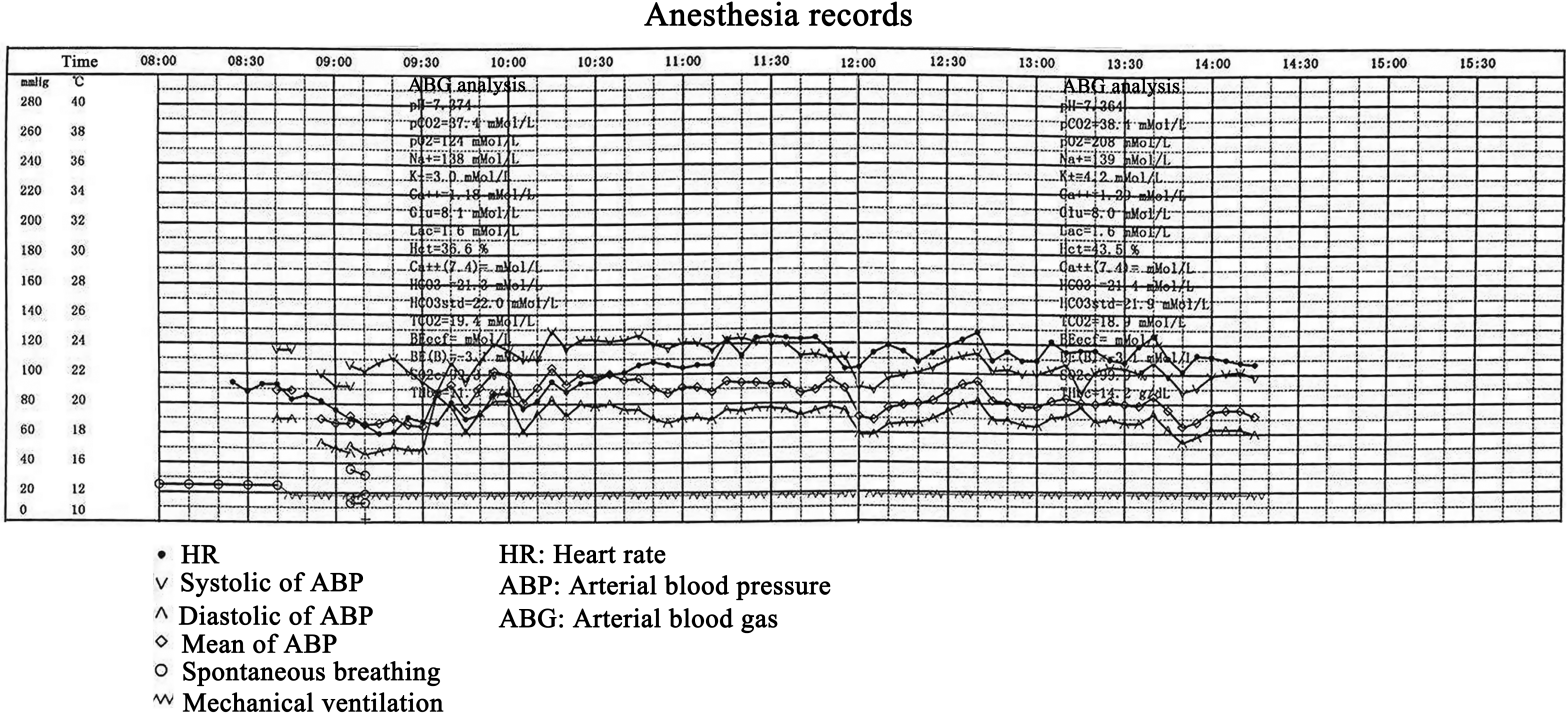
Anesthesia record of patient

Routine monitoring conducted after the patient entered the operation room showed the following vital signs: heart rate (HR), 88 beats/min; blood pressure (BP), 112/68 mmHg; respiration rate (RR), 20 breaths/min; and oxygen saturation (SpO2) level, 96%. Although awake fiberoptic intubation would have been ideal, it is not preferred in a frightened child. Initially, ketamine 10 mg and dexmedetomidine 10 μg were administered. After preoxygenation for 5 min, local anesthetic topicalization of the airway was followed by administration of lignocaine 10 mg and ephedrine 5 mg. Subsequently, successful intubation was achieved by fiberoptic intubation bronchoscopy via the nasal route to find the glottis, and a lubricated tracheal tube of internal diameter 5 mm was inserted through the nostril to enter the trachea. The breathing circuit was attached through an endotracheal tube connector, and adequate spontaneous ventilation was ensured. Further anesthetics were administered routinely, and the surgery proceeded smoothly with stable intraoperative vital signs (Figure 3).

After 2 days in the intensive care unit, the extubation was performed smoothly, and the patient was discharged to the rehabilitation ward with SpO2 at 97%. The patient was discharged from the rehabilitation ward after 8 days.

## 3 DISCUSSION

Yolk sac tumors, also called endodermal sinus tumors, are characterized by diverse clinical, pathologic, and prognostic features.^1^ YSTs generally exhibit poor prognosis, as they tend to recur locally, and a high incidence of metastasis.^2^ Therefore, therapy includes extensive surgical resection, intensive combinations of chemotherapy drugs, and occasional radiation therapy. However, patients who are subjected to general anesthesia after facial radiotherapy are at a significantly high risk of developing difficult airways. To the best of our knowledge, no cases of anesthesia induction in patients with facial YST have been reported. This report describes the anesthesia process for spontaneous ventilation bronchoscope intubation in a potentially difficult airway of a 7‐year‐old child with YST.

In the clinic, the prediction of a difficult airway requires comprehensive evaluation; however, there are certain characteristics that have been identified in patients requiring awake tracheal intubation (AKI), such as those with head and neck pathology (including malignancy, previous surgery, or radiotherapy), reduced mouth opening, limited neck extension, and progressive airway.^3^ AKI involves placing a tracheal tube in an awake, spontaneously breathing patient, usually with the help of flexible bronchoscopy or video laryngoscopy.^4^ This allows the airway to be secured before induction of general anesthesia, avoiding the potential risks and consequences when managing a difficult airway in an anesthetized patient. Because the patient in our case had undergone facial radiotherapy and systemic chemotherapy and had restricted mouth opening and limited head receding, there was a high risk of difficult airway intubation. Therefore, fiberoptic bronchoscopy was the technique of choice. In this case, a senior experienced anesthetist used fiberoptic flexible bronchoscopy for intubation while maintaining spontaneous breathing of the patient.

Moreover, because the patient's cooperation is essential for AKI, local anesthesia of the airway not only improves the child's acceptance of an airway device but also blocks the airway reflexes. Some studies have shown that awake fiberoptic intubation can be used in conjunction with either inhalational or intravenous induction in children.^5^ Esketamine 10 mg and dexmedetomidine 10 μg were administered in our patient for analgesia and sedation during anesthesia induction. Thus, their use during AKI can reduce patient anxiety and discomfort and increase procedural tolerance.

Another concern regarding difficult pediatric airways is the limited volume of topical local anesthesia that can be administered because of the small stature of the patient and the scarcity of data on the safe dosage of topical lidocaine. Our patient remained stable throughout the perioperative period. Comparing the risks of inadequate airway topicalization and of mild systemic lidocaine toxicity, combining intravenous induction and airway topicalization modalities seems favorable.

In conclusion, this is the first report of anesthesia in children with primary maxillofacial YST. Combining intravenous induction and airway topicalization modalities seems more favorable to the YST child with a potentially difficult airway.

## AUTHOR CONTRIBUTIONS

SZ, LW, HZ, and SL cared for patients and participated in writing and revising the paper. RD helped in manuscript revision. All authors have read and approved the manuscript.

## FUNDING INFORMATION

This project was funded by the National Natural Science Foundation of China (nos. 82103641, 81771354 and 81471106), the National Science Foundation of Hunan Province (nos. 2022JJ70061) from the Department of Anesthesia at Central South University. The funding body was not involved in the design, preparation, or writing of this manuscript.

## CONFLICT OF INTEREST

The authors declare that they have no competing interests.

## ETHICAL APPROVAL

The study was approved by the Research Ethics Committee of the second Xiangya Hospital, Central South University, Changsha, China.

## CONSENT

Written informed consent was obtained from the parent of the patient to publish this report in accordance with the journal's patient consent policy.

## Data Availability

All data related to this case report are contained within the manuscript.
